# A Flexible Approach for Assessing Functional Landscape Connectivity, with Application to Greater Sage-Grouse (*Centrocercus urophasianus*)

**DOI:** 10.1371/journal.pone.0082271

**Published:** 2013-12-13

**Authors:** Seth M. Harju, Chad V. Olson, Matthew R. Dzialak, James P. Mudd, Jeff B. Winstead

**Affiliations:** Hayden-Wing Associates LLC, Natural Resource Consultants, Laramie, Wyoming, United States of America; University of Western Ontario, Canada

## Abstract

Connectivity of animal populations is an increasingly prominent concern in fragmented landscapes, yet existing methodological and conceptual approaches implicitly assume the presence of, or need for, discrete corridors. We tested this assumption by developing a flexible conceptual approach that does not assume, but allows for, the presence of discrete movement corridors. We quantified functional connectivity habitat for greater sage-grouse (*Centrocercus urophasianus*) across a large landscape in central western North America. We assigned sample locations to a movement state (encamped, traveling and relocating), and used Global Positioning System (GPS) location data and conditional logistic regression to estimate state-specific resource selection functions. Patterns of resource selection during different movement states reflected selection for sagebrush and general avoidance of rough topography and anthropogenic features. Distinct connectivity corridors were not common in the 5,625 km^2^ study area. Rather, broad areas functioned as generally high or low quality connectivity habitat. A comprehensive map predicting the quality of connectivity habitat across the study area validated well based on a set of GPS locations from independent greater sage-grouse. The functional relationship between greater sage-grouse and the landscape did not always conform to the idea of a discrete corridor. A more flexible consideration of landscape connectivity may improve the efficacy of management actions by aligning those actions with the spatial patterns by which animals interact with the landscape.

## Introduction

Maintaining connectivity of landscapes for animal populations is a primary challenge for conservation and land managers [1], [2]. This challenge arises from two general sources: methodological and implementation. Methodological challenges include assumptions made about the nature of the system under study, and often include decisions implicitly based on our expectation of how animals should interact with the landscape, rather than modeling the ways in which animals exhibit functional interactions with the landscape. For example, previous methodological limitations have included basing connectivity models on expert opinion, treating the landscape as a binary classification of habitat vs. non-habitat, modeling structural connectivity of individual landscape components, and assuming animal pseudo-presence along straightline movement paths connecting known animal locations [3], [4], [5], [6], [7], [8], [9]. In particular, recent work [10] has overcome many of these challenges by looking at functional animal-environment relationships in a complex multivariate landscape based on known animal locations. However, a persistent methodological challenge that is not addressed in recent connectivity literature is that investigators (often unwittingly) use analytical methods that make the implicit assumption that connectivity is bestachieved via delineation of corridors. Addressing this methodological challenge may help overcome implementational challenges in applied connectivity management.

One of the primary challenges in implementation is that connectivity management is often addressed long after landscapes are heavily developed. For this reason, most connectivity work has focused on a corridor approach to maintaining connectivity. This approach has its own set of challenges (e.g., potentially ineffective design and cost-benefit tradeoffs [8], [11]), yet a more basic challenge is that animals often move through landscapes without regard for human-designated corridors [5]. Most methods for modeling connectivity implicitly assume the presence of discrete corridors (e.g., looking for the least-cost path between patches of habitat), essentially treating the landscape as patches of habitat in a sea of non-habitat [12]. This view is accurate in many conservation situations, such as landscapes that are already heavily developed (e.g., urban areas) or where a species’ ecology dictates a strict distinction between habitat and non-habitat (e.g., butterflies inhabiting meadows surrounded by willow thickets and coniferous forest, [3]). However, delineating discrete corridors may be unnecessarily limiting or unrealistic in other situations. Habitat generalists or highly mobile species may not necessarily use distinct corridors. For example, recent work [13] has documented altered resource selection of migrating mule deer (*Odocoileus hemionus*) in developed areas, although mule deer maintained high fidelity to a multitude of historic routes spread across the landscape. Even strict habitat obligates may not need corridors in landscapes with low to moderate levels of human modification. A generalized approach to modeling connectivity may also inform management of invasive species (often habitat generalists), a situation often considered outside of the realm of conservation-based connectivity research but one that may strongly benefit from applied management to reduce connectivity [14]. Stakeholders may resist implementing measures to maintain connectivity in a take-it-or-leave-it corridor network. Conceptualizing and investigating landscape connectivity as a generalized or potentially diffuse process, rather than as a series of rigid structural features such as corridors, would establish a more flexible framework from which to identify and conserve functional connectivity habitat. Addressing the methodological challenge of imposing spatial constraints on connectivity habitat may help overcome the largest challenges associated with implementation of connectivity management.

We used simple analytical techniques to test whether a species’ functional relationship with the landscape justified imposing a priori spatial constraints on connectivity habitat using a generalized connectivity modeling method that does not assume the existence of or need for discrete corridors. However, the approach we used will delineate discrete movement corridors if they are a natural pattern resulting from the functional relationship between animals and the landscape. We used the association between greater sage-grouse *Centrocercus urophasianus* (hereafter sage-grouse) and shrub-steppe habitat in western North America as a model system. Sage-grouse populations have experienced long term declines between 17 and 47% [15], currently occupy approximately 56% of their historic range [16], and have been designated warranted (but precluded due to higher priority species) for listing as threatened or endangered under the United States federal Endangered Species Act [17]. Connectivity is thought to be an important part of conservation and management in this system [18], [19]. Sage-grouse are a good case study for a generalized approach to modeling connectivity because they occur across a variety of shrub-steppe habitats, are considered a potential umbrella species for sagebrush steppe conservation [20] and exhibit both non-migratory and migratory movement between seasonal use areas within populations [21]. Energy development in the Intermountain West of the United States is occurring over large spatial scales and is currently the predominant expanding human use of the study area. Energy development, like other types of widespread human activity, has been shown to affect resource selection and population dynamics in sage-grouse [22], [23], [24] and through landscape-level modification of habitat may function to reduce connectivity of sage-grouse habitat via human activity associated with such development. Specific objectives of this work were to 1) use sage-grouse occurrence locations to infer latent movement states, 2) develop resource selection functions (RSF) for both sexes within each movement state, 3) generate maps predicting probability of occurrence across the landscape during moderate to long distance movement states (e.g., a connectivity map) and 4) validate the connectivity map using occurrence locations from independent sage-grouse in moderate to long-distance movement states.

## Materials and Methods

### Study Area

**Figure 1:**
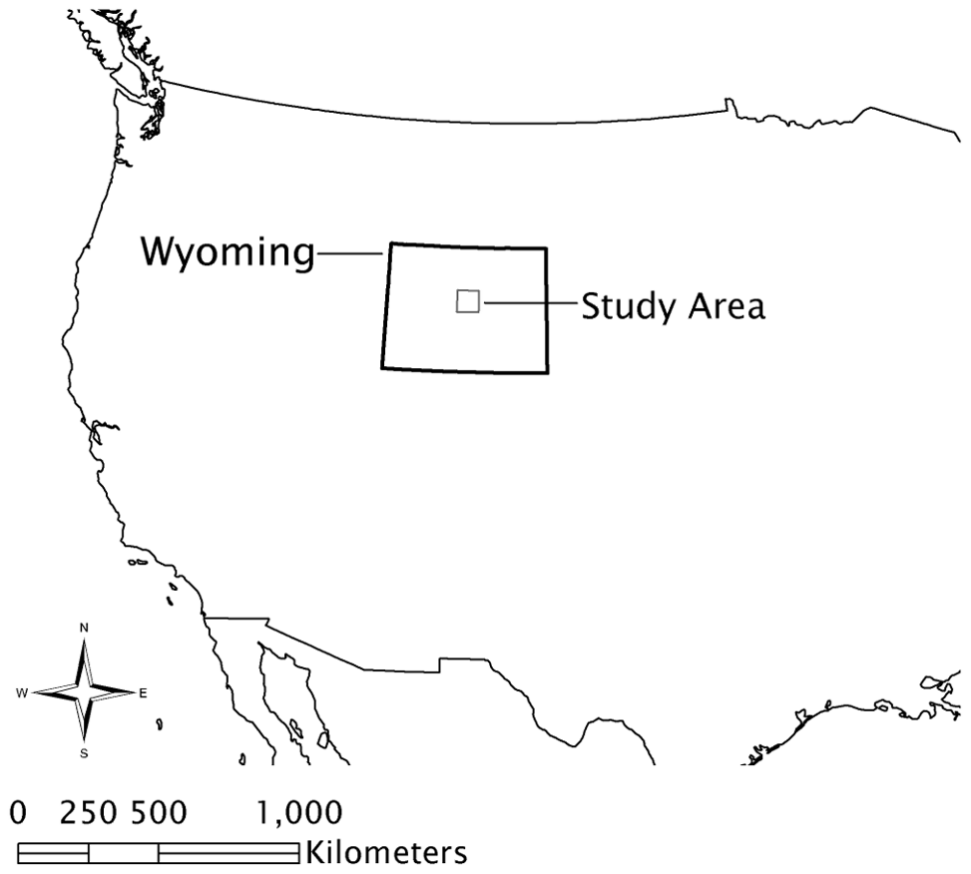
Map of study area boundary in central Wyoming, USA.

The 5,625 km^2^ study area included portions of the Wind River Basin in central Wyoming, USA (Fig. 1). Topography is variable with gently sloping flats, cut banks, dry washes, steep forested slopes and rocky canyons ranging in elevation from 1478–2776 m. In general, the southern half of the study area is a geographic basin and the northern half is characterized by increasingly steep slopes, valleys, and ridges. Minimum and maximum temperatures for each year during the study period were −29.3 and 36.7°C in 2008, −34.6 and 34.6°C in 2009, and −27.0 and 34.2°C in 2010; average total precipitation from three weather stations across the study site was 5.42 cm in 2008, 20 cm in 2009, and 17.9 cm in 2010 (C.V. Olson, unpublished data). Dominant plant species at lower elevation included Wyoming big sagebrush (*Artemisia tridentata wyomingensis*), basin big sagebrush (*A. t. tridentata*), black greasewood (*Sarcobatus vermiculatus*), winterfat (*Ceratoides lanata*) and shadscale (*Atriplex confertifolia*). At higher elevation, mountain big sagebrush (*A. t. vaseyana*), limber pine (*Pinus flexilis*), Douglas fir (*Pseudotsuga menziesii*) and rocky mountain juniper (*Juniperus scopulorum*) were present. The study area encompassed historic and ongoing development of energy resources. Oil and natural gas development was initiated in the 1920 s; gas development accelerated in the 1990 s. In 2010 there were 1,085 wells associated with oil and gas development in the study area.

### Field Procedures and Location Data

**Figure 2:**
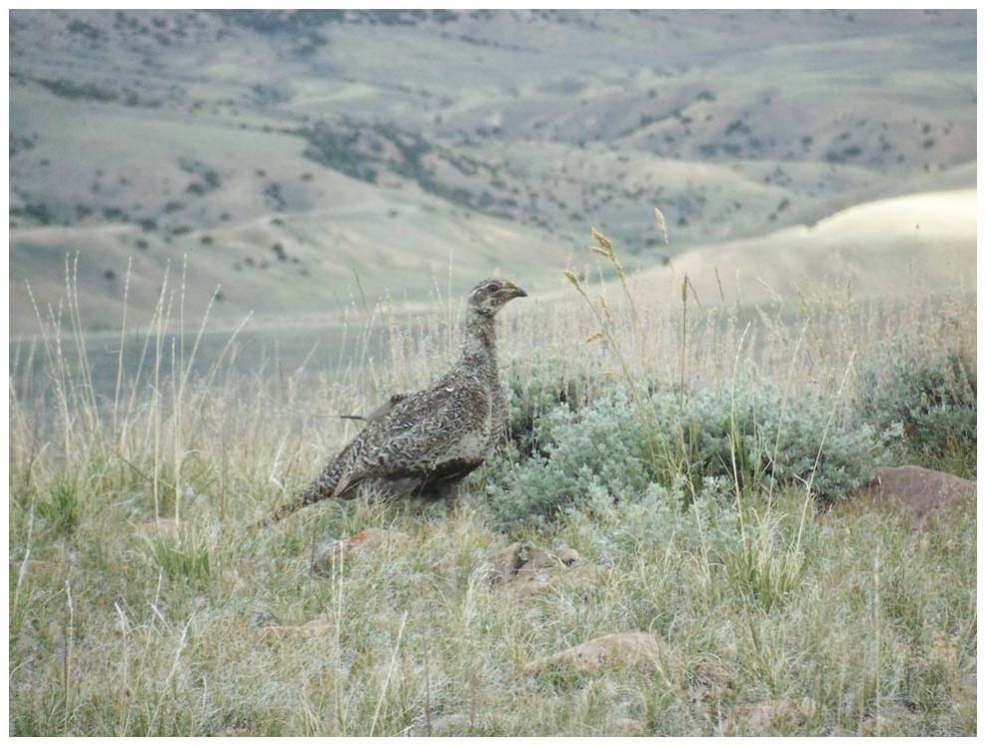
Female greater sage-grouse (*Centrocercus urophasianus*) in central Wyoming, USA, wearing rump-mount GPS unit. Photo credit T. Dorval.

From 2008–2010, we captured 42 male and 98 female sage-grouse by spotlighting [25] on and around leks dispersed throughout the study area, mostly during spring. Some captures occurred in summer and fall; in such cases capture effort was based on known location of other grouse to which GPS units were affixed. Permission to capture sage-grouse was granted by the Wyoming Game and Fish Department (Chapter 33 Permit #649), the relevant regulatory body concerned with protection of wildlife. The work was not approved by an Institutional Animal Care and Use Committee as all study design, animal capture and handling, and subsequent data collection and analysis was performed by employees of a private consulting firm. However, all animal capture and handling protocols were approved and conducted under a permit issued by Wyoming Game and Fish Department. We determined age and sex and fitted sage-grouse with 30-g ARGOS/GPS Solar PTTs (PTT–100, Microwave Telemetry Inc., Columbia, MD, USA). GPS units were attached using a rump-mount technique [26] (Fig. 2). GPS units had Ultra High Frequency (UHF) beacons for ground tracking and detection of mortality and had a 3-year operational life. Collars were programmed to record location information during 15 Feb–14 May every 3 h from 0700–2200, during 15 May–15 July every 1 hr from 0700–2100. During 16 July–31 Oct collars recorded location information every 3 hr from 1000–2200 and during 1 Nov–14 Feb every 6 h from 1000–2200. We did not include locations from female sage-grouse during the period when the individual was incubating eggs or caring for broods because in these cases movement behavior was constrained by distance from the nest or by slow movement capabilities of chicks. We had detailed field-based and GPS data on nest initiation, nest failure, hatch dates and brood fates for each adult female to determine excluded dates (C.V. Olson, unpublished data). We did not delineate the analysis by season because we viewed resource selection during long-distance movement as important during all seasons. Additionally, practical land management decisions would address comprehensive connectivity habitat needs (rather than only managing for fall connectivity habitat at the exclusion of spring connectivity habitat). Because we systematically collected and subsampled the GPS data throughout the course of each year, any seasonal differences were averaged out.

### Assigning Movement State

**Figure 3:**
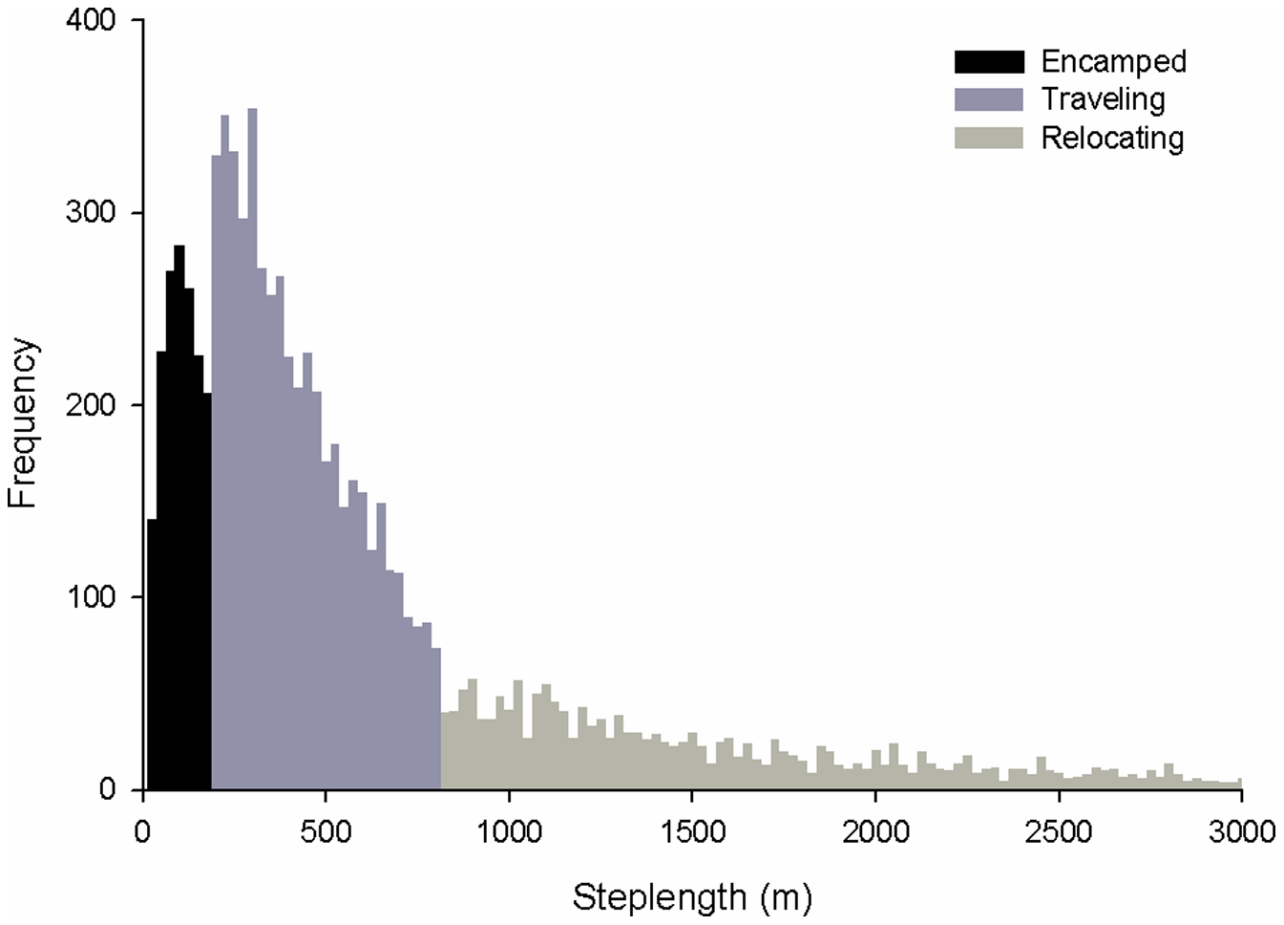
Histogram of 24-hr steplengths by greater sage-grouse in central Wyoming, USA, 2008–2010. Figure was right-truncated for display; longest 24-h steplength was 17,852 m. The movement states encamped, traveling and relocating were assigned to the shortest 25%, middle 50% and longest 25% of 24-hr steplengths, respectively.

We focused on three movement states for this analysis: encamped, traveling and relocating. We used 10 am locations for this analysis to remove diel variation in resource selection, to better capture long-distance movements, to model resource selection in relation to disturbance during the day time when human activity was most prominent and because preliminary analysis showed that distance between locations (steplength) was correlated with time between locations (thus requiring a consistent time interval between locations). Each location was assigned to a movement state based on distance from the 10 am location 24 hrs previous and distance to the next 10 am location 24 hrs hence. We discarded the first and last location from each individual because we did not have a previous or successive location from which to assign a movement state. Sophisticated methods to delineate movement states (e.g., [27], [28], [29]) did not work with our data set (S.M. Harju, unpublished data), perhaps because of differences in species ecology, temporal scales of data collection or the lack of distinct processes underlying separate movement states in sage-grouse [30], [31]. To approximate the ecological relationships between sage-grouse and resources in different latent movement states, we used a simple objective cutoff. We divided steplengths into three categories (Fig. 3) based on the distribution of distances between successive 24 hr locations (Males: mean = 732.44 m, median = 375.31 m, max = 17200.7 m; Females: mean = 738.49 m, median = 371.43 m, max = 19975.72 m). We assigned the shortest 25% of steplengths to the encamped movement state (<177.25 m Males; <168.81 m Females), the middle 50% of steplengths to the traveling movement state (>177.25 m and <800.38 m Males; >168.81 m and <798.87 m Females) and the longest 25% of steplengths to the relocating movement state (>800.38 m Males; >798.87 m Females).

Locations of sage-grouse are samples taken from unobserved movement states. To ensure confidence in the estimated movement state from which a location was sampled we retained only those locations with a consistent movement state immediately before and after that location. For example, location *l_1_* from a male sage-grouse was assigned to the movement state ‘relocating’ if the steplengths from *l_0_* to *l_1_* (24 hrs previous) and *l_1_* to *l_2_* (successive 24 hrs) were both >800.38 m. Although this resulted in discarding some location data (when we were unsure as to the movement state from which a given location was sampled), it provided a basis from which to objectively establish movement states from location data.

### Covariate Calculation

Using a Geographic Information System (GIS; ArcGIS® 9.3, ESRI, Redlands CA) and a 30 m grid cell size, we calculated covariates depicting landscape features that, based on field observation and previous research, influenced behavior of sage-grouse [21], [22], [32], [33]. We generated 7 covariates depicting predominant human modifications of the landscape (road density within 1 km and oil and natural gas well density within 1 km), landscape vegetation (mean percent coverage of sagebrush within an 800 m window, the standard deviation of the percent coverage of sagebrush across grid cells within an 800 m window, and the number of mesic grid cells within a 2 km window) and topographic features of the landscape (slope and terrain roughness [standard deviation of elevation] within 800 m). Raster images for oil or gas wells were developed using data provided by the Wyoming Oil and Gas Conservation Commission. Raster images of all other human modifications of the landscape were developed through heads-up digitizing of 2006 1-m resolution National Agriculture Imagery Program aerial imagery. We used Spatial Analyst in ArcGIS to calculate raster images and to extract values from raster data to location data for all covariates.

### Modeling Resource Selection

We used discrete choice models [34] to evaluate resource selection at spatial and temporal scales aligned with the underlying selection process during movement. Discrete choice models are suitable because: 1) they allow availability to be defined uniquely for each point, thus accounting for spatial constraints on availability due to short sampling intervals between successive locations, 2) they allow comparison of true use versus non-use, as an animal cannot simultaneously be at a used location and the paired random locations [35] and 3) they can capture the scale at which individuals perceive the environment and select resources during movement. Individuals likely have made an a priori decision to move in a given direction and thus are choosing locations from nearby combinations of landscape features. This is most important to consider during the traveling and relocating movement states as described above. During the encamped state discrete choice models reflect constrained availability as a function of limited movement behavior.

We matched each used location with a set of 50 non-used (random) locations that were available spatially and temporally but were not chosen. We prevented random locations from occurring within 30 m of each other to minimize pseudoreplication in sampling availability as a function of covariate raster grid cell size (30 m). We used a relatively large number of random locations to estimate selection of relatively uncommon variables, specifically those reflecting human modification of the study area (*sensu* [36]). Perception of availability among sage-grouse likely varied depending on the underlying movement state, thus we altered the size of the area from which available locations were drawn depending on movement state. For example, when relocating, it is plausible that individuals chose movement paths from a relatively large area, whereas when encamped individuals had already chosen to move short distances and thus had smaller areas that were functionally available to be selected. We sampled random locations within a 250 m radius buffer around encamped locations, a 1 km radius buffer around traveling locations and a 2 km radius buffer around relocating locations.

We used conditional logistic regression [37], [38] to estimate discrete choice models separately for each sex and each movement state. To test for collinearity among predictor variables we assessed pairwise Pearson correlation coefficients. All correlation coefficients were <0.75. We did not perform any model selection or variable reduction procedures and instead relied on the results from our parsimonious model for inference. We used program R for all statistical analyses (R Development Core Team, v. 2.13.2, 2011).

The strengths of this approach include: 1) it relies on known occurrence locations and an index of the movement path (because the true movement path is unknown), 2) it accommodates matrices of varying levels of habitat quality as well as binary habitat types, 3) is based on resource use decisions made by animals within the context of their movement state and perception of the landscape, and 4) allows, but does not assume, fundamentally different selection of resources during different movement states.

### Predictive Maps and Validation

We generated predictive probability of use maps by male and female sage-grouse during traveling and relocating movement across the study area. We calculated relative probability of use for each raster grid cell by inputting observed predictor variable values into the final model equation for each sex and movement state:
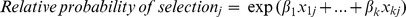
for each coefficient estimate (β) and observed value (*x*) for each predictor variable *k* at each grid cell *j*. The RSF value is unit-less, so to compare relative probability of use among the models we binned the RSF values within each model in 5 equal-sized bins (with values from 1 to 5) reflecting relative probability of occurrence in a grid cell ranging from low to high. We then summed the bin values for males and females while traveling and relocating (with values from 4 to 20). This map was then reclassified into 5 equal-sized bins to develop a general connectivity habitat map for all sage-grouse.

To validate this final predictive map we plotted the GPS locations from 20 sage-grouse (16% of all collared sage-grouse) that were withheld from the statistical analysis to evaluate how well the final connectivity habitat map predicted occurrence of independent sage-grouse during traveling and relocating movement states. We then performed Spearman rank correlation Chi-square tests to evaluate whether the difference in the number of observed versus expected locations increased monotonically with increasing probability of use bins.

## Results

Total sample sizes across movement states for model-building females and males were 5,125 and 2,251 locations, respectively (Table 1). We used 846 and 365 locations from independent female and male sage-grouse, respectively, for validation of the predictive connectivity map. The largest contribution of locations from a single sage-grouse to a model-building dataset was 20% (Male encamped). No other model-building dataset had a contribution from a single sage-grouse of more than 10% of the model-building locations (Table 1).

**Table 1:** Sample sizes for data used in model building and validation of the predictive connectivity map (travel + relocate) in central Wyoming, USA, 2008–2010.

	Sex	Movement state	# locations	# birds	Median #locns/bird	Min #locns/bird	Max #locns/bird
Model-building	Female	Encamped	913	73	8	1	54
		Travel	2909	78	25	1	136
		Relocate	1303	75	12	1	79
	Male	Encamped	397	33	6	1	79
		Travel	1244	34	27	1	107
		Relocate	610	31	17	1	61
Validation	Female	Travel	587	13	36	2	125
		Relocate	259	12	19	1	52
	Male	Travel	254	6	29	2	114
		Relocate	111	5	30	1	47

**Figure 4:**
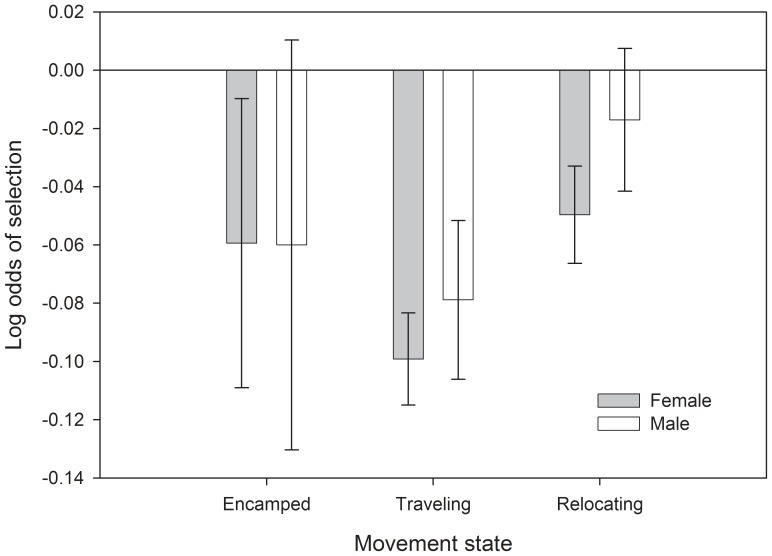
Resource selection by male greater sage-grouse in relation to topographic roughness during different movement states in Wyoming, USA. Topographic roughness was calculated as the standard deviation of elevation within an 800% confidence intervals.

**Figure 5:**
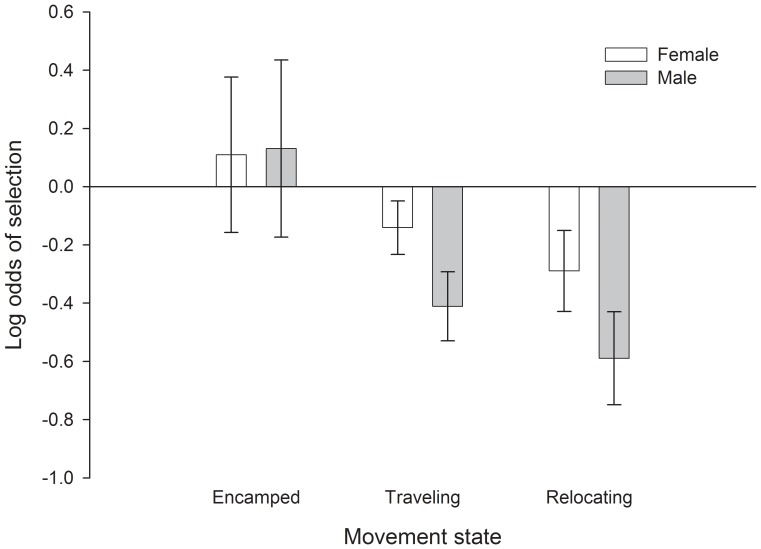
Selection for location in relation to natural gas well density (no. of wells within a 1 km window) by greater sage-grouse during different movement states. Error bars are 95% confidence intervals.

**Figure 6:**
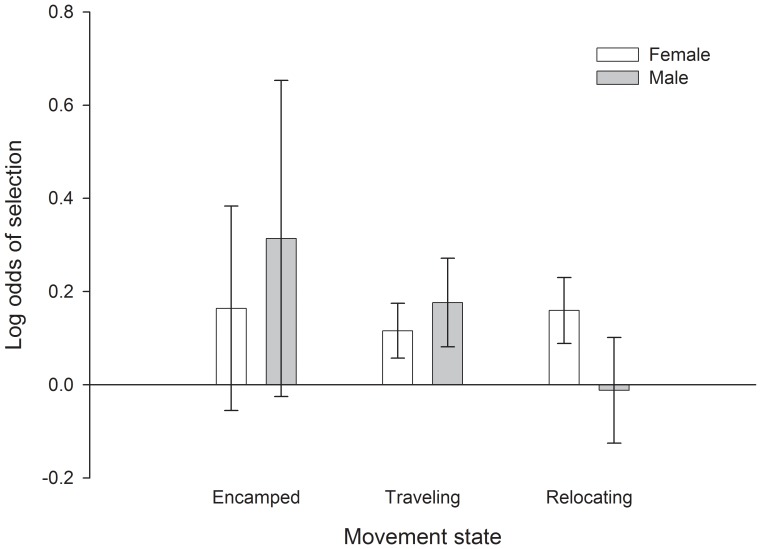
Selection for patchiness of sagebrush by greater sage-grouse in central Wyoming, USA. Patchiness of sagebrush calculated as the standard deviation of the percent coverage of sagebrush within each 30% confidence intervals.

Across sexes and movement states, sage-grouse tended to avoid areas with steep slopes and high topographic roughness (Fig. 4, Table 2). They often occurred in areas of higher road density but lower natural gas well density than available, especially for males that were traveling or relocating (Fig. 5). Both males and females selected for locations with a higher proportion of sagebrush as well as areas with greater patchiness of sagebrush (Fig. 6). One reviewer questioned whether avoidance of topographic roughness was stronger than the apparent selection for higher road density. We calculated x-standardized coefficient estimates (i.e., x-std(β_i_) = β_i_ * SD(x_i_)) to compare the strength of selection/avoidance for these variables, given that they have different units of measurement. The x-standardized coefficient estimates indicated that selection for higher road density was as strong as or stronger than avoidance of topographic roughness for males, whereas female avoidance of high topographic roughness was an order of magnitude stronger than their mild selection for higher road density (Table 3). The pairwise correlation coefficient between road density and topographic roughness was −0.26 for all non-used locations (non-used locations are an objective assessment of underlying spatial correlation of landscape feature values). See Table S1 for detailed results and Table S2 for summary statistics of predictor variables at ‘non-used’ locations for each movement state.

**Table 2:** Odds ratios of selection for predictor variables during different movement states by greater sage-grouse in central Wyoming, USA, 2008–2010.

		Predictor variable						
Sex	Movement state	Mesic areas^a^	Slope^b^	Topographic roughness^c^	Road Density^d^	Well Density^e^	Sagebrush^f^	Patchiness of sagebrush^g^
Female	Encamped	1.000	**0.929**	**0.942**	1.360	1.116	1.002	1.178
	Traveling	1.000	**0.939**	**0.906**	1.063	**0.868**	**1.147**	**1.123**
	Relocating	**0.999**	**0.947**	**0.952**	1.059	**0.749**	**1.115**	**1.173**
Male	Encamped	1.001	1.006	0.942	1.670	1.140	1.048	1.369
	Traveling	0.999	**0.951**	**0.924**	**2.417**	**0.663**	**1.088**	**1.193**
	Relocating	1.000	**0.940**	0.983	**1.945**	**0.555**	**1.127**	0.988

^a^ No. of mesic grid cells w/in 2.01 km window.

^b^ Degres.

^c^ Std. dev. of elevation (m) w/in 810 m window.

^2^.^d^ Total length (km) w/in 1 km

^2^.^e^ No. w/in 1 km

^f^ Percent sagebrush w/in 810 m window.

^g^ Std. dev. of percent sagebrush w/in 810 m window.

% CI does not overlap 1.0. See Table S1 for detailed results. Bold values indicate estimates where 95

**Table 3:** Standardized coefficient estimates of selection for topographic roughness and road density by traveling and relocating greater sage-grouse in central Wyoming, USA, 2008–2010.

Sex	Movementstate	Topographicroughness^a^	Roaddensity^a^
Female	Traveling	−0.958	0.035
	Relocating	−0.427	0.034
Male	Traveling	−0.653	0.657
	Relocating	−0.111	0.524

β_i_) = β_i_ * SD(x_i_).^a^ X-Standardized coefficient calculated as: X-std(

**Figure 7:**
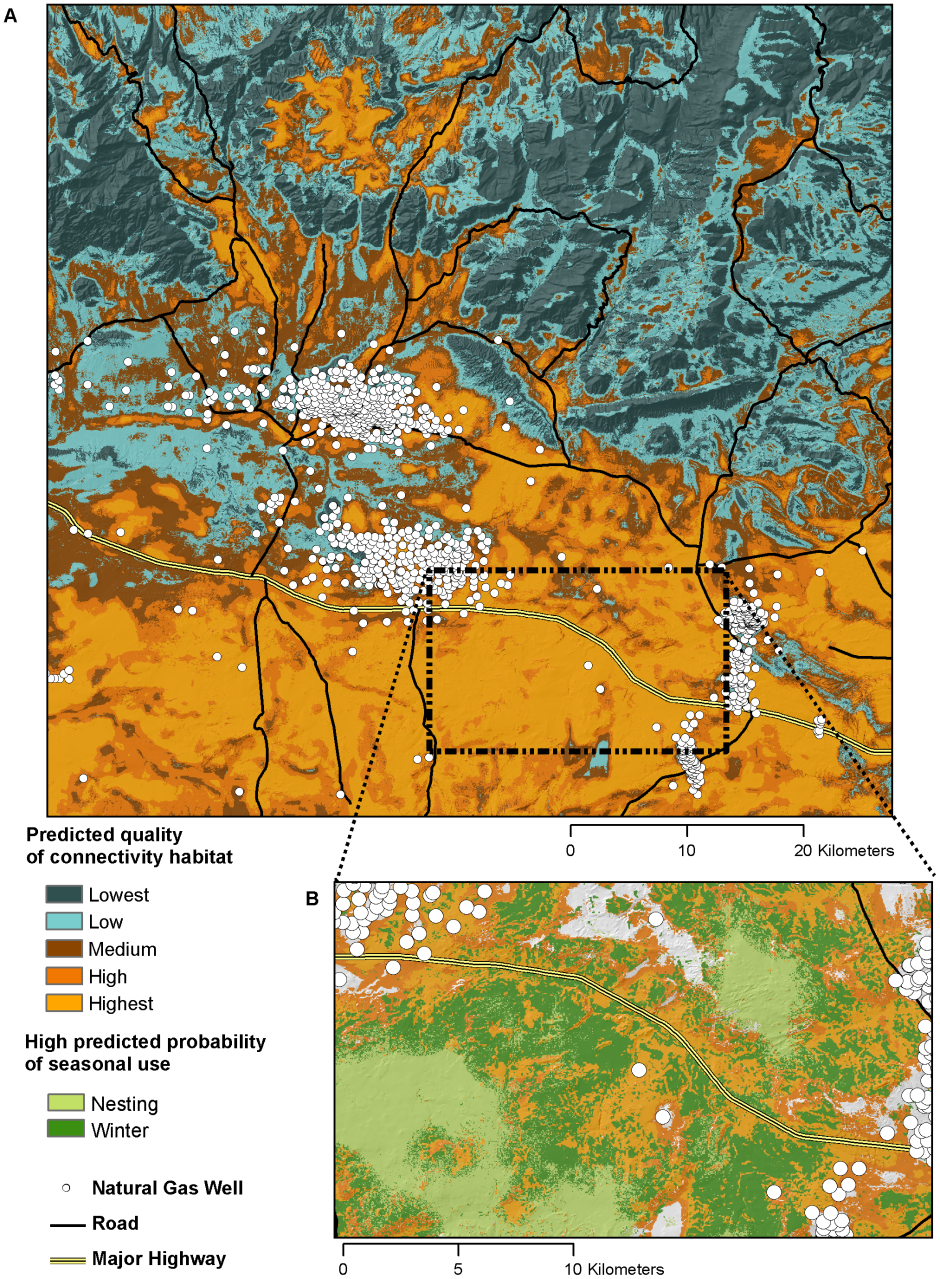
Map of connectivity habitat quality for greater sage-grouse in central Wyoming, USA. Panel ‘a’ is the entire study area. Panel ‘b’ illustrates a practical application of the map where critical seasonal use layers are overlaid on top of the two highest connectivity habitat quality layers. The nesting [33] and winter [34] layers are from companion analyses conducted on the same population of sage-grouse during the same time period.

Discrete connectivity corridors were largely absent from most of the study area, where either high or quality connectivity habitat occurred across broad areas. However, in the northwest quarter of the study area, flat valley bottoms appeared to function as distinct corridors of high quality connectivity habitat (Figure 7a).

**Figure 8:**
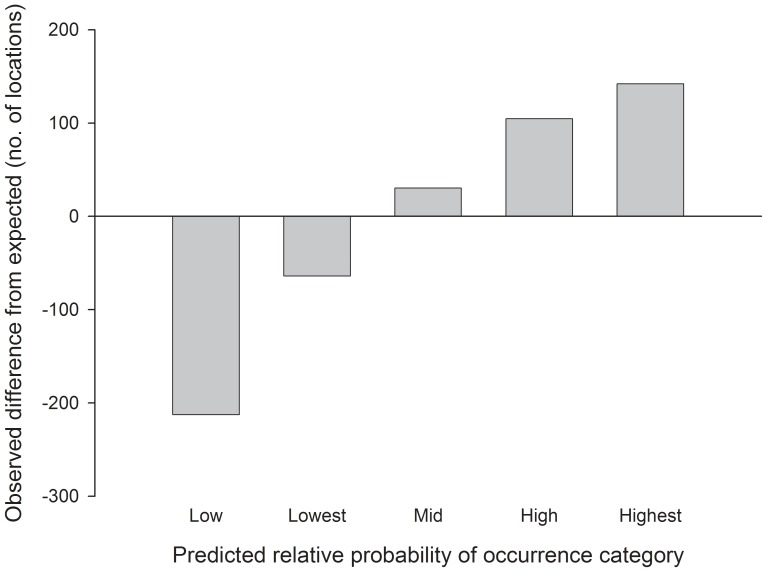
Difference in the number of observed versus expected independent greater sage-grouse GPS locations central Wyoming, USA. Fewer observations than expected (negative values) and more observations than expected (positive values) in lower and higher use categories, respectively, indicate the connectivity model performed well at predicting occurrence and resource selection of independent greater sage-grouse. Only locations from traveling and relocating movement states of independent birds were used for validation.

The 1,211 validation locations (Table 1) fell more often in high predicted probability of use areas and less often in low predicted probability of use areas than expected at random (Spearman’s rho = 1.0, 3 d.f., p<0.001; Fig. 8). This indicates that the combined connectivity map (traveling + relocating, both female and male; Fig. 7a) performed very well at predicting the occurrence of independent male and female sage-grouse during traveling and relocating movement states.

## Discussion

Traditionally, connectivity modeling has assumed the presence of discrete corridors as a de facto component of species or community ecology or as a requisite of land use planning in human-modified areas [5], [8], [12]. A general feature of the final connectivity map (Fig. 7a) is that distinct ‘connectivity corridors’ were largely lacking, although they did appear to occur in some areas. A second feature of the connectivity map is that it conceptually treats connectivity habitat as a spatially variable feature of the landscape. This conceptual treatment is an underpinning of empirically assessing the functional relationships between animals and the landscape [10], [39]. Human assessments of the configuration and value of landscapes are inherently faulty, as they do not fully incorporate perception distances, the animal’s weighting of co-occurring landscape features, and the presence of behavioral syndromes among mobile animals [39], [40]. Methods that rely on empirical data overcome this challenge. Approaches to connectivity modeling that do not impose spatial constraints (e.g., least cost path analyses explicitly assume, a priori, that the desired outcome is linear) increase the flexibility of the modeling approach to accurately reflect the functional relationship between animals and the landscape. Flexibility increases the potential for success of connectivity management actions and allows for connectivity management to be integrated with other management priorities.

The utility of this approach for maintaining connectivity of animal populations is strengthened by integration with GIS [33], [41], [42], [43]. Here, we used the final resource selection models for traveling and relocating male and female sage-grouse to develop a comprehensive ‘connectivity’ map, essentially identifying areas likely to be selected or avoided by sage-grouse when they are moving moderate to long distances (Fig. 7a). This map can be used to maximize the return on conservation and management dollars by identifying areas where enhancements or restrictions would most benefit sage-grouse, minimize unnecessary restrictions on development by identifying areas where restrictions would have minimal benefit to sage-grouse connectivity and ultimately increase the positive benefit to sage-grouse populations under multiple land use scenarios by simultaneously increasing stakeholder buy in to management plans, maximizing the effectiveness of management actions, and increasing the spatial extent of areas to be conserved. In our case study, the spatially-explicit connectivity map could be used to ensure landscape connectivity between other areas important to sage-grouse, such as critical nesting, brood-rearing or winter habitat (Fig. 7b; [33], [44]), possibly by delineating areas of high density of high quality connectivity habitat [8]. Connectivity maps could also be combined with spatially-explicit models of mortality risk to identify attractive sinks where managers may want to discourage connectivity [32], [33], [45]. An alternative application would be modeling connectivity of undesirable animal populations (e.g. invasive species) to identify areas where management actions would be most effective at reducing connectivity of populations..

The approach we used overcomes many shortcomings of alternative methods of modeling connectivity for non-corridor species, although it has its own limitations. We set different scales of availability for each movement state, which has been noted to make comparisons among analyses difficult [46]. We did this because it seems reasonable that an individual animal’s perception of availability is related to its underlying movement state and maintaining a single scale of availability across movement states could generate results inconsistent with the underlying biological process of resource selection [47]. Thus we accepted potential sampling errors in order to gain potential ecological reality. There are two tests of these potential limitations. The first is to compare the landscape to determine if what we characterize as ‘available’ resources is systematically influenced by our scale of defining what is available [46]. We found that the characterization of the landscape was similar for each movement state, especially given wide variation in observed values for each of our predictor variables (Table S2). The second test is in the validation of the predictive RSF models. The final sage-grouse connectivity map performed well at predicting occurrence of independent sage-grouse during traveling and relocating movement states, suggesting that any methodological limitations did not extensively hamper inference and application.

These results also offer general inference and specific application to managing connectivity for the focal species greater sage-grouse. Landscape-level connectivity of shrub-steppe habitat has been identified as critical to maintenance of sage-grouse populations [48], [49], [50], [51], [52]. The general assumption has been that managers need to delineate corridors (e.g., [51], [52]). In this study, there was general lack of distinct corridors due to the spatial configuration of sage-grouse connectivity habitat within the study area. What characterized high quality connectivity habitat mirrors the factors that numerous studies have identified as high quality sage-grouse habitat during critical times of year, such as nesting, brood-rearing, and wintering (e.g., [22], [33], [53]). Specifically, we also documented avoidance of natural gas and oil wells, steep slopes, and areas of rough topography and a preference for higher coverage (and patchiness) of big sagebrush. Perhaps surprisingly, we also documented a preference for portions of the landscape with higher density of roads. This is the opposite pattern observed in a separate analysis we performed on winter habitat selection by this same population of sage-grouse, where we documented avoidance of roads during both day and night [44]. It is important to clarify that sage-grouse (particularly males) selected for locations that had higher road density than was available to them at that time and place rather than what was available at the landscape level. A low landscape-level correlation between road density and topographic roughness, combined with an equal strength of selection for these variables by male sage-grouse (Table 3), indicates that male selection of higher road density when traveling and relocating was a real pattern in the study area. We therefore hypothesize that perhaps road density does not act as a barrier to connectivity even though roads may be negatively associated with occurrence during particular seasons. This finding highlights the value of an empirical approach to modeling connectivity, as an expert-based assessment of connectivity habitat would have likely considered high road density as a barrier to movement. Using this generalized conceptual and analytical approach to modeling landscape connectivity allows for empirical-based models that do not constrain the spatial pattern of connectivity into corridors, unless corridors are a natural part of how animals interact with the landscape.

## Supporting Information

Table S1 **Full model results.**
(XLS)

Table S2 **Summary statistics of predictor variables at ‘non-used’ locations for each movement state.**
(XLS)
